# The *Notch-2* Gene Is Regulated by Wnt Signaling in Cultured Colorectal Cancer Cells

**DOI:** 10.1371/journal.pone.0017957

**Published:** 2011-03-18

**Authors:** Jonas Ungerbäck, Nils Elander, John Grünberg, Mikael Sigvardsson, Peter Söderkvist

**Affiliations:** Division of Cell Biology, Department of Clinical and Experimental Medicine, Faculty of Health Sciences, Linköpings university, Linköping, Sweden; University Medical Center Hamburg-Eppendorf, Germany

## Abstract

**Background:**

Notch and Wnt pathways are key regulators of intestinal homeostasis and alterations in these pathways may lead to the development of colorectal cancer (CRC). In CRC the *Apc/β-catenin* genes in the Wnt signaling pathway are frequently mutated and active Notch signaling contributes to tumorigenesis by keeping the epithelial cells in a proliferative state. These pathways are simultaneously active in proliferative adenoma cells and a crosstalk between them has previously been suggested in normal development as well as in cancer.

**Principal Findings:**

In this study, *in silico* analysis of putative promoters involved in transcriptional regulation of genes coding for proteins in the Notch signaling pathway revealed several putative LEF-1/TCF sites as potential targets for β-catenin and canonical Wnt signaling. Further results from competitive electrophoretic mobility-shift assay (EMSA) studies suggest binding of several putative sites in Notch pathway gene promoters to *in vitro* translated β-catenin/Lef-1. Wild type (wt)-Apc negatively regulates β-catenin. By induction of wt-Apc or β-catenin silencing in HT29 cells, we observed that several genes in the Notch pathway, including *Notch-2*, were downregulated. Finally, active Notch signaling was verified in the *Apc^Min/^* ^+^ mouse model where *Hes-1* mRNA levels were found significantly upregulated in intestinal tumors compared to normal intestinal mucosa. Luciferase assays showed an increased activity for the core and proximal *Notch-2* promoter upon co-transfection of HCT116 cells with high expression recombinant Tcf-4, Lef-1 or β-catenin.

**Conclusions:**

In this paper, we identified *Notch-2* as a novel target for β-catenin-dependent Wnt signaling. Furthermore our data supports the notion that additional genes in the Notch pathway might be transcriptionally regulated by Wnt signaling in colorectal cancer.

## Introduction

The epithelium of the gastrointestinal tract is continually replaced with a turn-over rate of two to seven days. In order to maintain homeostasis of the intestinal epithelium, processes cellular proliferation, differentiation, migration and death must be strictly regulated [1]–[3]. A few but highly conserved signaling pathways are thought to drive these processes (reviewed in [4], [5] and [6]). The canonical Wnt signaling pathway was the first to be discovered as being essential for intestinal crypt cell proliferation and homeostasis [7]–[10]. One central component of this pathway is the cytoplasmic protein β-catenin, which when translocated into the nucleus as a result of Wnt signals, serves as a co-factor for transcription factors of the Lef-1/Tcf (Lymphoid enhancer factor-1/T Cell Factor) family to allow for activation of a downstream genetic program [11]. The level of β-catenin in the colon epithelium is regulated by the ubiquitinin-proteasome system [12]. One of the critical components of the β-catenin destruction complex is the adenomatous polyposis coli (Apc) protein [11]. Mutational inactivation of this gene causes stabilization of β-catenin [13] and increased cell proliferation and represents one of the most common genetic alterations in colorectal cancer (CRC) [14]. This results in increased levels and nuclear translocation of β-catenin and subsequent dysregulated activation of LEF-1/TCF target genes [5].

The maturation of intestinal stem cells is also regulated by the Notch signaling pathway representing another evolutionary conserved signaling system involved in maintaining colon epithelium homeostasis [2]–[4], [15]–[17]. Core elements in this signaling pathway are the monomeric transmembrane bound Notch receptors (Notch1–4 in mammals), which upon binding to ligand (Deltalike-1, -3, -4, Jagged-1 or -2) release an intracellular domain (NICD) that serves as a transcriptional co-factor. The specificity of ligand/receptor interaction is determined through addition of sugar moieties by the glycosyltransferases from the *Fringe* gene family (*Lunatic fringe*, *Lfng*; *Maniac fringe*, *Mfng* and *Radical fringe*, *Rfng*) [18]–[20]. NICD translocates into the nucleus to form a transcriptional activation complex with the DNA-binding factor CSL (Rbp-jκ) and co-activators belonging to the *Mastermind-like* family (*Maml*) [15], [21], [22]. Some of the best-characterized targets of this transcriptional activation complex belong to the *Hes* -and *Hey* family of genes, which function as transcriptional repressors of further downstream targets like *Math-1* (mouse homolog of human *Hath-1*) [23]–[25]. It has been suggested that Notch-1 and Notch-2 function redundantly in the gut, and that canonical pathway activation through either of these receptors is sufficient to prevent differentiation of proliferative crypt progenitor cells into post-mitotic goblet cells, indicating that Notch signaling could be predisposing for malignant transformation [2]. This has been associated with derepression of the cyclin-dependent kinase inhibitors p27^Kip1^ and p57^Kip2^ [2] as well as upregulation of *Math-1* mRNA and protein [2], [26], [27]. However, *Notch-2* has also been proposed to have a tumor suppressive effect in CRC [28], suggesting complex, possibly stage related, functions of Notch signaling in the intestine.

In addition to the independent roles of Wnt and Notch signaling pathways in tumorigenesis in the colon, the findings that tumor development in Apc-deficient mice is enhanced upon simultaneous activation of Notch and Wnt signals [29] and that many intestinal tumors display abnormal activation of both pathways [3] suggest a molecular interplay between Notch and Wnt signaling in the formation of CRC. Despite the apparent importance of this crosstalk and that coordinated actions have been reported at different levels [30], little is known about the molecular mechanisms linking these pathways in the intestinal epithelium. It has been shown that *Hath-1* expression is increased when the Wnt pathway is inhibited [26] and that *Hes-1* is a direct target of canonical Wnt signaling in colorectal adenomas and carcinomas [29], [31] Furthermore, Jagged-1 has been shown to represent a molecular link between Wnt and Notch in CRC, where the *Jagged-1* gene is directly regulated by β-catenin/Tcf-4 [32]. The interactions between Wnt and Notch in CRC, and the results thereof, are not fully understood and the results from different studies are often diverging [3], [31]. This emphasizes the complexity of the crosstalk and suggests that it may not simply be a matter of shared downstream targets of the pathways.

In this study, we have investigated the potential of the Wnt signaling pathway for direct regulation of genes involved in Notch signaling. This revealed that several of the putative promoter regions in Notch pathway associated genes contain Lef-1/Tcf-binding sites. Some of these genes were downregulated upon expression of a functional Apc gene in the HT29 CRC cell line, supporting the idea that abnormal Wnt signaling has a direct impact on the expression of genes encoding proteins involved in Notch signaling. Thus, we propose that crucial components of the Notch signaling pathway are directly influenced by Wnt signaling with the implication that even a genetically normal Notch pathway can contribute to tumorigenesis due to the response to β-catenin or *Apc* mutations.

## Results

### Promoter sets and *in silico* identification of potential LEF-1/TCF-sites in Notch pathway promoters

**Figure 1:**
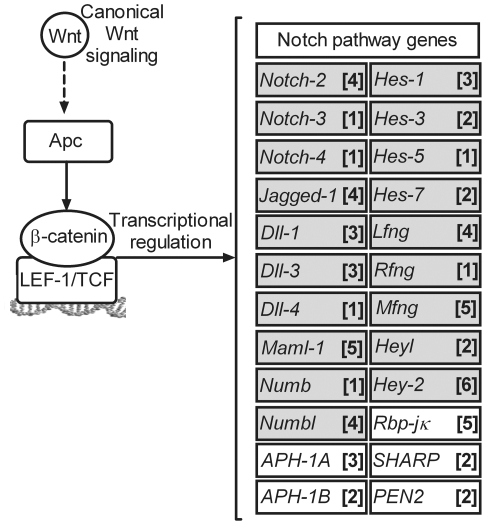
*In silico* analysis of Notch pathway gene promoters. *In silico* analysis of the genetic networks directly involved in Notch and Wnt signaling suggest overlap and direct crosstalk via Notch target gene activation through canonical Wnt signaling. By means of MatInspector, [33] gene promoters in the Notch pathway were found to contain at least one putative LEF-1/TCF-site (number of sites in per gene is described in square brackets). Genes in gray boxes were subjected for further semi quantitative RT-PCR analysis.

To identify potential targets for interactions between the Notch and Wnt signaling pathways, 65 genes, known to be important for Notch and Wnt signaling, were selected bioinformatically using the Ingenuity Pathways Analysis (Ingenuity® Systems, www.ingenuity.com), together with extensive literature searches. Gene promoter sequences were extracted using the Genomatix Gene2Promoter software and the average length of the putative core, proximal and parts of the distal promoters were adjusted to approximately 2500 bp (details available on request). By means of the MatInspector software [33] the promoter sets for putatitve LEF-1/TCF sites were identified with the putative consensus sequences: 5′-(A/T)(A/T)CAA(A/T)G-3′ [34]. Twenty-four of the investigated genes in the Notch pathway were found to contain one or several putative LEF-1/TCF sites (Fig. 1 and Supplemental Information S1).

Thus, *in silico* analysis of the genetic networks involved in Notch and Wnt signaling supports the previously shown interactions with *Hes-1* [31] and *Jagged-1* [32] and, furthermore, suggests a direct crosstalk via target gene activation on several additional levels in the Notch pathway.

### The β-catenin/Lef-1-complex binds in vitro to Notch pathway gene promoters

To determine whether the *in silico* identified LEF-1/TCF binding sequences physically bind to the β-catenin/Lef-1 complex *in vitro*, we conducted a competitive electrophoretic mobility-shift assay (EMSA) with a radioactively labeled consensus LEF-1/TCF strong binding probe (CD1TOP [35]) and duplex oligonucleotides covering the potential sites in target promoters. Notch pathway EMSA promoter probes (Table S1) were tested against an *in vitro* translated Lef-1/ß-catenin complex.

**Figure 2:**
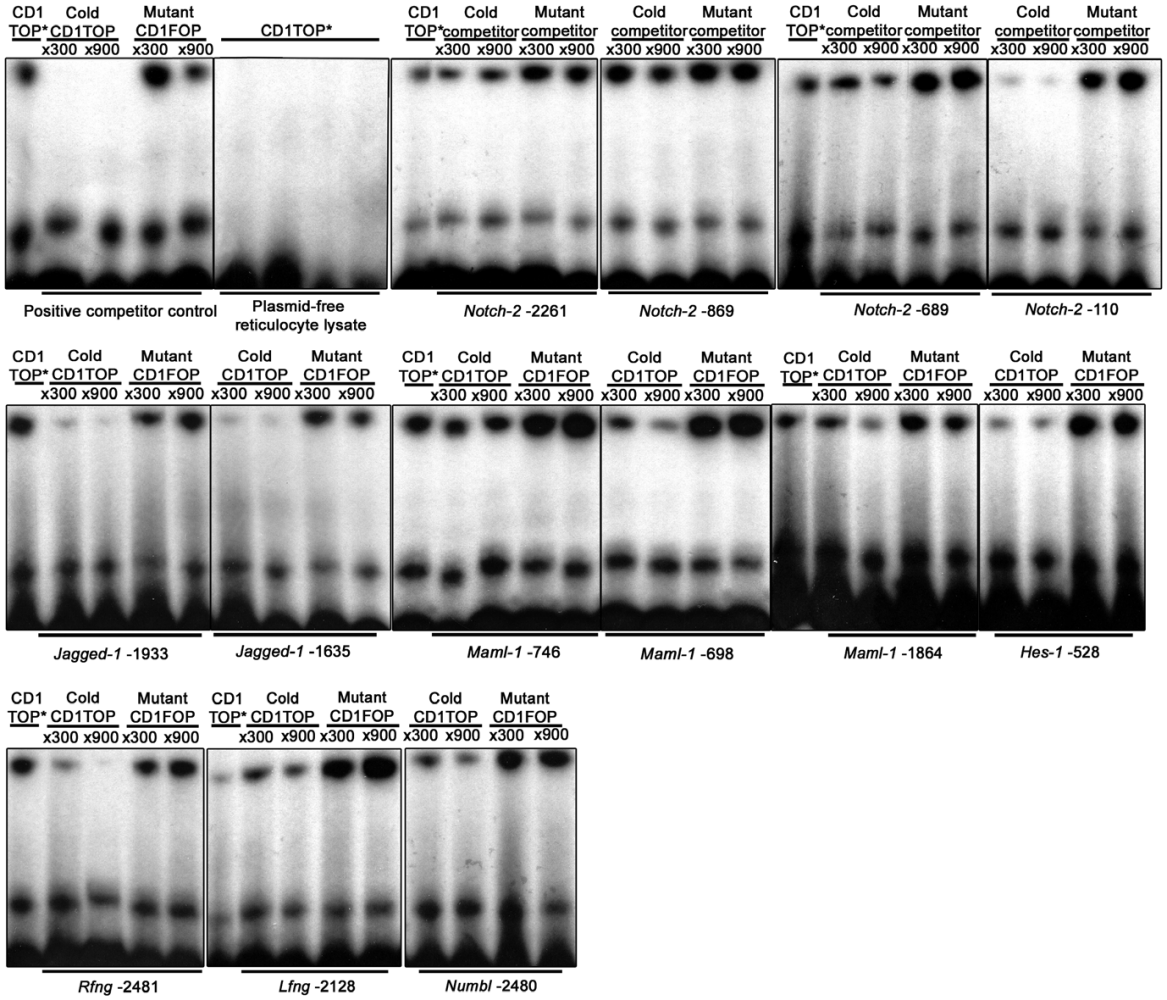
*In vitro* translated β-catenin/Lef-1 binds to Notch pathway gene promoters. Competitive electro mobility-shift assay of the proximal *Notch-2* promoter. Duplex CD1TOP probes were “end labeled” with [^32^P]dATP, incubated with *in vitro* translated β-catenin/Lef-1 and exposed to competition with abundance of cold or cold mutated promoter duplex oligonucleotides (×300 and ×900, respectively). The protein-DNA complexes were separated by electrophoresis and visualized by autoradiography. As a competition control cold CD1TOP and cold mutated CD1FOP competed with radiolabeled CD1TOP and to confirm that radioactive labeled CD1TOP binds Lef-1 specifically, plasmid-free reticulocyte lysate were subjected to *in vitro* translation and incubated with radiolabeled probe. Gene numbering describe position of the LEF-1/TCF-site relative the gene translation start site. Adjustments in whole image contrast levels were performed in Adobe Photoshop CS4.

To confirm that radioactively labeled CD1TOP binds Lef-1 specifically, plasmid-free reticulocyte lysates were subject to *in vitro* translation and incubated with a radiolabeled probe. As expected, no binding was observed (Fig. 2). All of the investigated *Notch* gene promoters, *Notch-2*, *Jagged-1*, *Maml-1*, *Hes-1*, *Rfng*, *Lfng* and *Numbl*, showed binding of at least one putative LEF-1/TCF site in their promoter regions identified *in silico* (Fig. 2) (additional competitive EMSA results are found in ). To our knowledge *Notch-2*, *Maml-1*, *Rfng* and *Lfng* are new potential Wnt target genes not described previously. Two of the strongest binding sites were found in the *Jagged-1* promoter at −1933 and −1635 relative to the translation start site, which is in accordance with earlier studies where *Jagged-1* has been shown to be a β-catenin/Tcf-4 regulated gene in human CRC [32] as well as in mouse hair follicles [36]. *Hes-1* has recently also been identified as a direct transcriptional target of β-catenin/Tcf-4-dependent Wnt signaling in CRC [31] and our EMSA data supports this notion by clearly identifying *Hes-1* -528 as a binding site for the β-catenin/Lef-1 complex (Fig. S1).

The results from both the *in silico* and DNA binding analyses suggest that a transcriptional network links the Wnt and Notch pathway, implying a functional regulation of canonical Wnt signaling at several levels of the Notch pathway.

### Canonical Wnt signaling regulates Notch pathway genes in HT29 colorectal cancer cell line and murine intestinal adenomas

**Figure 3:**
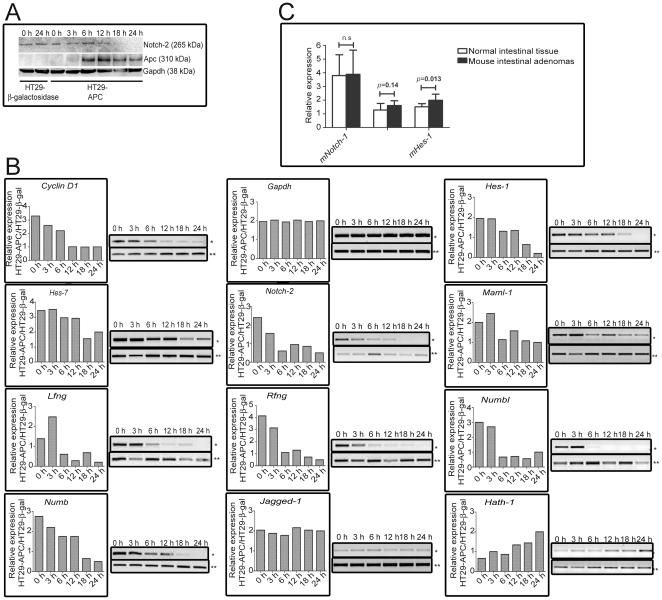
The expression of *Notch* pathway genes are dependent of *Apc* status in HT29 cells and murine intestinal adenomas. Wt-Apc was induced in HT29-APC cells through the addition of 100 µM ZnCl_2_ to the growth medium. Semi-quantitative RT-PCR was carried out on cDNA reversely transcribed from 200 ng total RNA from each time point (0–24 h post wt-Apc induction). Bars describe the relative expression of *Notch-2* in HT29-APC cells (*) versus HT29-β-galactosidase cells (**) normalized against *Gapdh* expression. *Cyclin D1* was used as a positive control for Wnt inactivation. (B) The protein expression of Apc (∼310 kDa), Notch-2 (∼265 kDa), wt and loading control, Gapdh, was determined with Western blot following 0–24 h of zinc stimulation. Adjustments in whole image contrast levels were performed in Adobe Photoshop CS4. (C) Relative mRNA expression of murine *Notch-1*, *Notch-2* and *Hes-1* in tumors and corresponding non-tumor normal intestinal mucosa of Apc^Min/+^ mice. mRNA expression was related to the endogenous control gene GAPDH. White columns (n = 16), black columns (n = 22). Bars are presented as median expression values. Error bars describe interquartile range.

Mutational inactivation of the *Apc* gene is a key event in colorectal carcinogenesis and renders constitutive active Wnt signaling [13], [14]. In order to develop the *in silico* and DNA binding analyses and functionally investigate the effects of activated Wnt signaling on Notch pathway genes, nineteen genes in the Notch pathway, known to be important for canonical Notch signaling (Fig. 1 and Table S1), containing putative LEF-1/TCF-sites, were selected for further studies with semi-quantitative RT-PCR. To activate wt-Apc and thereby restricting the levels of nuclear β-catenin, a HT29 cell line carrying a Zn-inducible wt-Apc vector was used. As expected, there was an increase of wt-Apc levels in HT29-APC, 6–24 h post Zn^2+^-stimulation, but not in HT29-β-gal cells (Fig. 3A). The 19 Notch pathway genes were analyzed by semi-quantitative RT-PCR and *Hes-1*, *Hes-7*, *Notch-2*, *Maml-1*, *Lfng*, *Rfng*, *Numb* and *Numbl* were found to be transcriptionally downregulated, while the negatively regulated gene *Hath-1* was clearly upregulated 18–24 h post wt-Apc induction (Fig. 3B). As a positive control, the *cyclin D1*-gene expression, induced by β-catenin [37], was downregulated 6–24 h post Zn-induction, confirming inhibition of the Wnt pathway via expression of wt-Apc and reduced β-catenin levels. The results are in concordance with previous studies where β-catenin protein levels have been found to be downregulated [38] and the β-catenin/hTcf-4 nuclear complex reduced [39] upon Zn-stimulation in HT29-APC cells indicating, decreased levels of nuclear β-catenin. Interestingly, we could not detect any influence of Wnt signaling and wt-Apc expression on *Jagged-1* in HT29 cells even though a confirmed specific binding of CD1TOP to *in vitro* translated Lef-1 was displaced with a 300-fold excess of cold *Jagged-1* −1933 and −1635 probes but not with their mutated variants (Fig. 2). To determine whether downregulation of the Notch pathway genes, by wt-Apc, are directly under control of β-catenin, we performed anti-β-catenin siRNA silencing experiments (Fig. S2), using a pool of four different siRNAs targeting β-catenin. In general, downregulation was less significant than Zn induced decrease in *Notch-2*, *Numb and Numbl* (Fig. 2A), displaying a weak but consistent decrease in their respective mRNA levels. *Hes-1* was clearly downregulated 72–96 h post transfection, corroborating the findings by Peignon *et* al. [31]. *Rfng* and *Lfng* were not affected by β-catenin silencing indicating that their downregulation by Wnt signaling observed in Fig. 3B may not be a direct effect of β-catenin induced transcription used in this experimental setup. *Cyclin D1* was used as a positive control for the siRNA experiments and was clearly downregulated 72–96 h post transfection. To test more long-term effects of deregulated Wnt signaling in intestinal epithelium we analyzed *Notch-1*, *Notch-2* and *Hes-1* mRNA expression levels in Apc^Min/+^ mice (Fig. 3C) carrying a germline truncating heterozygous mutation at codon 850. The mRNA levels of *mHes-1* were found to be significantly upregulated in adenomas (median relative expression = 2.0, interquartile range = 1.5–2.4) compared to normal intestinal mucosa (median relative expression = 1.5, interquartile range = 0.9–1.7) (Mann-Whitney U-test, *p* = 0.013), indicating overactivated Notch signaling in the mouse adenomas. *A* trend towards upregulation was observed for *mNotch-2* in tumor tissue (median relative expression = 1.6, interquartile range = 1.0–2.0) compared to normal intestinal mucosa (median relative expression = 1.3, interquartile range = 0.9–1.8) (Mann-Whitney U-test, *p* = 0.14), while no statistically significant differences could be detected for *mNotch-1* (median relative expression = 3.9, interquartile range = 2.5–5.7 (tumor tissue)) vs. (median relative expression = 3.8, interquartile range = 2.4–5.3 (normal tissue)) (Mann-Whitney *U*-test, *p* = 0.9).

In conclusion, inactivation of the Wnt pathway through activation of wt-Apc in HT29 CRC cells downregulates several target genes in the Notch pathway while mRNA-levels of the Notch target *mHes-1* is significantly upregulated in intestinal tumors from Apc deficient mice. This further supports a direct transcriptional crosstalk suggested by the *in silico* and *in vitro* DNA-binding analyses.

### The *in silico* identified Notch-2 promoter contains four putative LEF1/TCF-sites and contributes to a high luciferase gene activity

Notch-2 functions redundantly with Notch-1 in colon epithelial cells where both genes are important for keeping the cells in the crypt compartment in a proliferative and undifferentiated state [2]. However, relatively little is known about transcriptional regulation of the *Notch* genes and a potential regulation of *Notch-2* through Wnt signaling could be of importance in the development and/or progression of CRC and potentially in other malignancies as well. Previously, it has been shown that both *Hes-1* and *Jagged-1* are direct targets of canonical Wnt signaling in CRC [31], [32]. Our results from *in silico*, *in vitro* DNA-binding analyses and activation of wt-Apc, strongly suggest that there is a direct positive regulation of *Notch-2* through Wnt-signaling. Therefore we wanted to further elucidate the β-catenin/Lef-1/Tcfs regulating potential of the *Notch-2* promoter in the CRC cell lines HT29 and HCT116.

**Figure 4:**
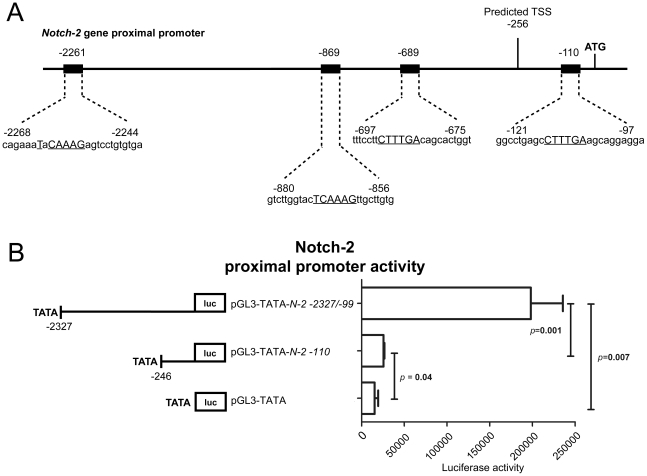
The *Notch-2* promoter contains four putative LEF1/TCF-sites and results in high luciferase gene activity. (A) Schematic representation of the proximal *Notch-2* promoter with the four putative LEF-1/TCF-binding consensus sites, identified with Genomatix MatInspector, at positions −2261, −869, .689 and −110 relative translational start site (ATG). A potential transcription start site was mapped to position −256 using Genomatix Gene2Promoter and PromoterInspector software. Uppercase letters indicate the core consensus sequence. (B) N2PR −2327/−99, N2PR −110 in pGL3-TATA as well as empty pGL3-TATA (Smith *et al*, 2002 [40]) were transfected into HCT116 and and co-transfected with pSV-β-galactosidase control vector. The cell lysate 24 h post transfection was subjected to luciferase reporter assays and relative luciferase activity determined. Error bars describe SEM.

As described above, *in silico* analyses were used to identify the human *Notch-2* promoter and a potential transcriptional start site (TSS) was found 256 bp upstream the translational start site, verifying the result from Gene2Promoter analysis. Four putative LEF-1/TCF consensus sites were identified in the core and proximal promoter region at positions −2261, −869, −689 and −110 relative to the translational start site (Fig. 4A). The −110 site showed strong binding to Lef-1 in the *in vitro* DNA binding competitive EMSA assay while sites −2261 and −689 showed weak binding (Fig. 2).

To analyze the *Notch-2* promoter, we focused on the 2200 nucleotide region spanning both the 5′ and 3′ regions of the *Notch-2 putative* transcriptional start site. Two separate constructs were generated, one spanning from position −2327 to −99 (N2PR −2327/−99) (numbers relative to translational start site), thereby covering all four putative LEF-1/TCF-sites and one covering putative LEF-1/TCF-site −110. They were cloned into a firefly luciferase reporter vector (pGL3 Luciferase reporter vector, Promega) containing a TATA-box to detect possible enhancer and repressor *cis*-acting elements (described previously [40]). The constructs were transiently transfected into HCT116 or HT29 cells and reporter gene activities were measured at 24 hours post transfection. Promoter activity was enhanced 13-fold for N2PR −2327/−99 vs. 1.7-fold for N2PR −110 promoter construct compared to the background in HCT116 cell lines (p<0.007 vs. p = 0.04, n = 6 (Fig. 4B)). The luciferase assay shows that the cloned region of the putative *Notch-2* promoter contains enhancer elements leading to enhanced transcription of the luciferase gene. These results strongly suggest the location of the core, the proximal part and parts of the distal *Notch-2* promoter as identified by the *in silico* analysis.

### Overexpression of β-catenin, Lef-1 or Tcf-4 result in increased Notch-2 promoter activity

*Notch-2* mRNA and protein levels are clearly downregulated 18–24 h post Zn-induction of wt-Apc in HT29-cells (Fig. 3B and B). *Notch-2* mRNA expression was also analyzed following *RNAi* silencing of β-catenin in HT29 cells where a weaker effect was observed similar to the other wt-Apc/β-catenin regulated Notch pathway genes (Fig. S2).

**Figure 5:**
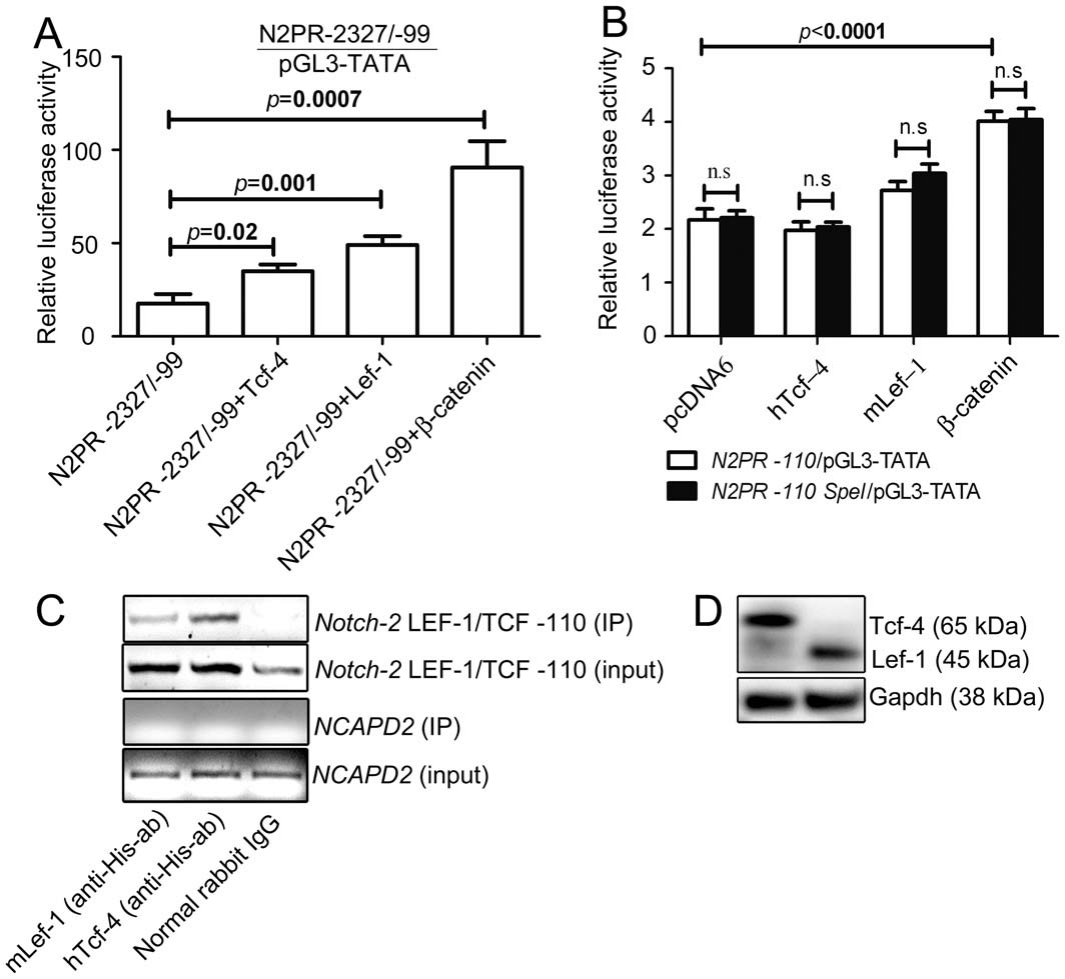
The *Notch-2* promoter is transcriptionally activated via Lef-1/Tcf-4/ß-catenin mediated signaling. (A) HCT116 cells were co-transfected with pGL3-TATA carrying N2PR −2327/−99 (or empty pGL3-TATA) and HA-S33Y-β-catenin in pCGN, hTcf-4 or mLef-1 in pcDNA6 as well as pSV-β-galactosidase control vector (n = 6). (B) HCT116 cells were co-transfected with pGL3-TATA carrying N2PR −110 (white columns) or mutated N2PR −110 SpeI (black columns) (or empty pGL3-TATA) and HA-S33Y-β-catenin in pCGN, hTcf-4 or mLef-1 in pcDNA6 as well as pSV-β-galactosidase control vector. Luciferase activity was normalized against pGL3-TATA background activity by dividing N2PR −2327/−99-pGL3-TATA relative expression with the relative expression from empty luciferase vector (n = 9). Error bars describe SEM. (C) ChIP in HCT116 cells co-transfected with His-tagged Lef-1 or Tcf-4, N2PR −2327/−99 and N2PR −110; immunoprecipitation with anti-His (6× His tag® antibody) or IgG control, PCR with primers encompassing LEF-1/TCF-site −110 in the *Notch-2* promoter. A 172-bp segment of *NCAPD2* promoter was used as a negative control. (D) Western blots with anti-His (6× His tag® antibody) verifying the transfection of His-tagged Lef-1 (∼65 kDa) and Tcf-4 (∼45 kDa) in HCT116. Adjustments in whole image contrast levels were performed in Adobe Photoshop CS4.

The results from the HT29-APC cell line where Apc was activated or β-catenin silenced, imply an activating role of the Wnt pathway on *Notch-2* and the downstream target gene *Hes-1* [31]. Therefore, we predicted that overactivation of canonical Wnt signaling through β-catenin/LEF-1/TCF would result in increased *Notch-2* promoter activity. To further elucidate the activation and interactions suggested by the Wnt inactivation experiment, we subjected HCT116 cells to luciferase assays using the N2PR −2327/−99 and N2PR −110 promoter constructs. To study if Wnt signaling effects on *Notch-2* are mediated through β-catenin/Lef-1/Tcf, cells were co-transfected with high expression vectors (pCGN and pcDNA6) containing S33Y-β-catenin (S33Y mutation), hTcf-4 or mLef-1 sequences and the relative luciferase activity was calculated by normalizing *Notch-2* promoter activity (N2PR −2327/−99 or N2PR −110) vs. pGL3-TATA-activity. Western blots confirmed expression of His-tagged Tcf-4 and Lef-1 (Fig. 5D). Relative luciferase activity was significantly increased for co-transfection with S33Y-β-catenin, both for N2PR −2327/−99 as well as N2PR −110 with a fold increase of 5.2 for N2PR −2327/−99 (p = 0.007) and 1.8 for N2PR −110 (p<0.0001) compared to the pcDNA6 co-transfection (5A, B and Supplemental Information S1). The increase in N2PR −110 activity upon co-transfection with mutated β-catenin was confirmed in HT29 (1.6-fold, *p* = 0.03) (Fig. S3). The results imply that β-catenin has the ability to activate the core and proximal *Notch-2* promoter. Tcf-4 and Lef-1 also enhance the N2PR −2327/−99 activity construct (2.0 fold increase (Tcf-4) (p = 0.02) and 2.8 (Lef-1) (p = 0.001), respectively. However, the increase in activity was not as significant as that seen upon β-catenin co-transfection, indicating more specific effects of the DNA-binding proteins. Tcf-4 and Lef-1 failed to significantly affect the activity of N2PR −110 and no differences could be detected between wild-type and mutated construct, where the putative LEF1/TCF site at position −110 was mutated into a *SpeI* restriction site. These results suggest that LEF1/TCF consensus site −110 is not a direct target of canonical Wnt signaling in this system through Tcf-4 or Lef-1 (Fig. 5B). However, contrasting this, competitive EMSA at the *Notch-2* LEF-1/TCF −110 site displayed specific binding to *in vitro* translated β-catenin/Lef-1 (Fig. 2), where 300-fold excess of cold but not mutated −110 probe displaced the interaction between CD1TOP and *in vitro* translated Lef-1. *Notch-2* −2261 and −689 sites only competed weakly with CD1TOP binding to *in vitro* translated Lef-1 compared to their mutated variants and were these therefore excluded from the ChIP analysis. In addition to EMSA, we examined the DNA-protein binding between Lef-1 or Tcf-4 and the *Notch-2* promoter in HCT116 cells transfected with recombinant His-tagged Lef-1 or Tcf-4 by ChIP. To increase the sensitivity, cells were co-transfected with N2PR −2327/−99 and N2PR −110 luciferase constructs. Western blots with 6× His tag® antibody confirmed efficient transfection of His-tagged Lef-1 and Tcf-4 (Fig. 5D). Following immunoprecipitation and appropriate washing steps, harvested DNA was amplified as a short 93-bp amplicon of the *Notch-2* promoter covering LEF-1/TCF-site −110. We observed successful immunoprecipitation of both Tcf-4 and Lef-1 (Fig. 5C), somewhat contradicting the results from the site-directed mutagenesis and subsequent luciferase experiment of site −110. A 178-bp fragment of the *NCAPD2* promoter was used as a negative control and showed no immunoprecipitation (Fig. 5C).

Overall, these results suggest that *Notch-2* can be transcriptionally regulated by canonical Wnt signaling, however the exact mechanism and interactions sites remain to be elucidated.

## Discussion

Wnt and Notch signaling pathways are both important regulators of intestinal homeostasis and play major roles in the development of CRC [2]–[4], [7]–[11], [14], [15], [17]. They have been shown to have synergistic effects in intestinal cell fate decisions and overactivation of Notch signaling in *Apc* deficient mice results in earlier onset and more tumors in the small intestine, compared to *Apc* deficient mice alone. However, overexpression of Notch signaling does not by itself cause intestinal tumor formation in the mouse [16]. Incremental evidence suggests a crosstalk between the two pathways [36], [41]–[48] although detailed knowledge regarding their transcriptional interaction network is relatively scarce. The results of Notch and Wnt interactions in intestinal adenomas are complex and most likely context dependent. van Es *et* al. [3] have demonstrated that blockage of Notch in the context of overactive Wnt signaling leads to the differentiation of proliferative mouse intestinal tumors into post-mitotic goblet cells and reduced tumor growth, something Peignon *et al*. [31] failed to corroborate in a conditional model where both the *Apc* and *RBJ-κ* genes were deleted simultaneously, the latter reflecting a model of more acute Apc loss. In the present study, *in silico* analysis of Notch and Wnt pathway gene promoters resulted in the identification of several putative β-catenin regulated LEF-1/TCF-sites in Notch pathway gene promoters and RBP-Jκ-sites in Wnt pathway gene promoters (Fig. 1 and Supplemental Information S1). Tissue-specific gene expression can be predicted from the nucleotide sequence approximately 1–2 kb upstream (and partly downstream) TSS [49], and therefore the length of the promoters, further investigated with the MatInspector software were adjusted to approximately 2500 bp. Both Lef/Tcfs and Rbp-jκ have strong DNA-binding domains and transcription is activated upon binding to β-catenin or NICD, respectively [50]–[54]. Twenty-four of the investigated gene core and proximal promoters in the Notch pathway contain at least one putative LEF-1/TCF consensus site and *Hey-2* contains as many as six while 28 of the bioinformatically investigated genes in the Wnt pathway contain one or more RBP-Jκ-sites (Supplemental Information S1). Recently, Rodilla and co-workers [32] performed a study where they blocked Wnt signaling through β-catenin in Ls17T CRC cells using a dominant negative (dn) Tcf-4 inducible vector and identified transcriptional targets using microarray mRNA expression profiling and quantitative RT-PCR. Interestingly, *Jagged-1* was identified as one of the most clearly affected targets and transcriptionally activated by the β-catenin/Tcf-4-complex. The interaction was verified *in vivo* thereby establishing Jagged-1 as a pathological link between Wnt and Notch pathways in CRC. However, to the best of our knowledge, the putative LEF1/TCF-binding sites in *Jagged-1* responsible for this interaction are unknown. The *in silico* determined core, proximal and parts of the distal *Jagged-1* promoter contains four putative LEF-1/TCF-sites. In this study, we show that the β-catenin/Lef-1 complex is capable of binding to the *Jagged-1* promoter *in vitro* at positions −1933, −1635 and possibly −1083 relative to the translation start site (Fig. 2 and Fig. S1) making these plausible sites for transcriptional interactions between *Jagged-1* and the canonical Wnt pathway. However, upon activation of wt-Apc in HT29 CRC cells, no effects on *Jagged-1* mRNA levels were detected (Fig. 3B), which could be a result of different cell lines and study design. In addition to *Jagged-1*, *in vitro* translated β-catenin/Lef-1 was found to bind LEF-1/TCF-sites in *Notch-2*, *Maml-1*, *Hes-1*, *Rfng* and *Lfng* gene promoters. Of these genes *Hes-1* has been shown to be an important direct target of ß-catenin/Tcf-4 mediated Wnt signaling in CRC [31], which our data confirms. A HT29 (lacking wt-Apc) cell line carrying a Zn-inducible wt-Apc [39] vector was used to regulate the β-catenin levels and usually wt-Apc was detected with Western blot analysis after 6 h of zinc stimulation (Fig. 3A), compared to a similar HT29 cell line carrying a Zn-inducible β-galactosidase vector.

Using semi-quantitative RT-PCR, we found *Notch-2*, *Maml-1*, *Hes-1*, *Hes-7*, *Rfng*, *Lfng*, *Numbl*, *and Numb*, as well as controls like *cyclin D1* and *Hath-1* to be transcriptionally up/downregulated after Zn-induced Apc expression (Fig. 3B). As expected *Hath-1* mRNA levels were upregulated synchronously with *Hes-1* downregulation, further supporting Wnt signaling effects on Notch –and Notch downstream mediators. Several of these genes may be regulated through canonical Wnt signaling but upon silencing of β-catenin, the downregulation of expected target genes was less significant compared to the wt-Apc induction, which may be explained by incomplete inhibition by the siRNA treatment (Fig. S2A). It is also possible that the kinetics is different for a direct β-catenin inhibition compared to wt-Apc induction, where several additional factors like mRNA-degradation and stability may affect the mRNA levels. In order to investigate this future studies are warranted. In accordance with the results of Peignon *et al*. [31] *Hes-1* mRNA levels were found upregulated in intestinal tumors from Apc deficient mice compared to normal intestinal mucosa. The authors also reported significantly higher levels of *Notch-1* and *Notch-2* in the murine tumors, something we failed to corroborate even though *Notch-2* mRNA levels possibly follow this trend (Fig. 3C). Differences might be explained by the use of different models. Their mice mainly develop colonic polyps whereas the Apc^Min/+^ model mostly generates tumors in the small intestine. Apc^Min/+^ mice are heterozygous for the *Apc* gene, which may lead to heterogenous β-catenin activation, which might obscure the effects on the Notch target genes.

*Notch-2* mRNA and protein levels were found to be downregulated upon wt-Apc induction in HT29 cells (Fig. 3B and B) and results from β-catenin siRNA silencing implied that Wnt signaling, at least partially, affects *Notch-2* via the β-catenin/LEF-1/TCF gene target program (Fig. S2A). Since *Notch-1* and *Notch-2* have been shown to have redundant roles in the intestine [2] a potential dysregulation of *Notch-2* through disrupted Wnt signaling could be of importance in the development and progression of CRC. Contrasting this, Chu *et al.* [28] found that *Notch-2* expression may play a role in tumor inhibition in CRC where Notch-2 protein and mRNA levels were found to be decreased and that high levels correlates with differentiation of colon cancer cells. Recent evidence shows that overactive Notch signaling is an important early event in human colorectal carcinogenesis where the pathway is overactivated in cancer-initiating cells and early adenomas [32], [55], [56] compared to a lower activity in more advanced or metastatic CRCs [29]. It is therefore not unlikely that *Notch-2* is deleted in advanced cancer stages but still plays an important role in tumor formation. *Notch-1* lacks putative LEF-1/TCF consensus sites in the proximal promoter, where *Notch-2* contains four LEF-1/TCF sites, which may suggest a direct canonical Wnt-dependent regulation of *Notch-2* mRNA levels.

We therefore asked whether canonical Wnt signaling could increase the *Notch-2* promoter activity. HT29 cells are hard to transfect and we therefore used a similar cell line, HCT116, which instead of inactive Apc contains a mutated and constitutively active β-catenin gene. Luciferase assays indicate an increased activity for N2PR −2327/−99 upon co-transfection with mutated β-catenin, Tcf-4 or Lef-1 (Fig. 5A). N2PR −110 only responded to β-catenin and the results from the site-directed mutagenesis imply that β-catenin affect the *Notch-2* core promoter via non-canonical Wnt signaling. It is previously known that β-catenin can interact directly with NICD [45], [47], [48] and that *Notch-1* is autoregulated in mouse lymphocytes [57]. The proximal *Notch-2* promoter investigated in this study also contains two RBP-Jκ-sites, indicating a potential autoregulatory mechanism similar to *Notch-1* regulation. In a preliminary study N2ICD but not N1ICD was found to overactivate the *Notch-2* promoter in a luciferase assay (unpublished data). Speculatively, β-catenin may interact with N2ICD to potentiate the expression of *Notch-2*, but more studies are needed to outline this interaction. Contrasting this, EMSA assays suggested binding of Lef-1 to the −110 LEF-1/TCF-site (Fig. 2), which also could be verified with immunoprecipitation where, in addition to recombinant Lef-1, recombinant Tcf-4 also successfully precipitated this part of the promoter region (Fig. 5C). This might suggest that Tcf-4 or Lef-1 have the ability to bind the promoter region where site −110 is located if the conditions are satisfied. However if this is the site for interaction between Wnt signaling and *Notch-2* in CRC is uncertain, and likely a larger part of the promoter, and thereby the additional binding sites suggested by the *in silico* and EMSA analyzes, are needed.

Both *Tcf-4* and *Tcf-1* are expressed in the intestinal mucosa [58], while the cells in the crypts of colon express only *Tcf-4* [7], [59]. *Lef-1* is normally not expressed in the intestinal tract but expression has been detected in CRC and several CRC cell lines, together with *Tcf-4* and *Tcf-1* [7], [58]. This suggests that alterations on several levels in the canonical Wnt pathway may contribute to overactive Notch signaling, possibly both through mutational inactivation of *Apc* and/or overexpression of other components in the Wnt pathway.

Briefly, we also studied whether the Wnt pathway (*i.e* β-catenin) was transcriptionally regulated by the NICD/Rbp-jκ complex, and thereby indicating a regulatory feed-back loop, through β-catenin. By γ-secretase treatment all Notch signaling is effectively inhibited and in our experiments the well-known Notch target gene *Hes-1* [60], was almost completely downregulated (Fig. S4). Previous studies, have established Wnt/β-catenin signaling as a negative target of Notch signaling, *e.g.* through upregulation of *Sfrp1*, *Sfrp5*, *Dkk1*, which are known to encode inhibitors of the Wnt pathway [43], [61]–[63]. In our HT29 cell line model, preliminary results cannot confirm that β-catenin is a Notch target in CRC cells since we do not see any effects on β-catenin or its target *cyclin D1* by DAPT treatment (blocking of intracellular Notch signaling) (Fig. S4A and B). These results are in close agreement with a study by Fre *et al.* [16] where the expression of Tcf-4 and Lef-1 were found to be unaffected by Notch pathway activation in mouse intestine, further indicating Notch-independent activation of canonical Wnt signaling. Also, if β-catenin had been negatively regulated by Notch in the intestinal tract, inhibition would rather lead to increased number of intestinal polyps. Active Notch signaling, however, clearly acts oncogenically in this context where Notch inhibition can lead to goblet cell formation and a decreased number of polyps [29], [64].

In conclusion, in this paper we identify several potential target genes of Wnt/β-catenin signaling among genes traditionally classified as belonging to the Notch pathway. Analysis of the genetic networks directly involved in Notch and Wnt signaling suggests functional overlap and direct crosstalk via target gene activation. More specifically, we suggest that *Notch-2* is a novel target, activated by Wnt signaling in colon cancer cells.

## Materials and Methods

### *In silico* identification of LEF-1/TCF-sites in Notch pathway gene promoters

Identification of potential promoter regions and putative LEF-1/TCF-sites therein was performed with Genomatix software (http://www.genomatix.de, Genomatix Software GmbH, Munich, Germany) [33]. The Gene2Promoter software (Models: library Vertebrate_Modules Version 4.5) was used to retrieve and identify promoters approximately within 2500 bp upstream the first exon of each retrieved gene (complete list is available on request). The highest quality sequence obtained was then used in the MatInspector software (MatInspector Release professional 7.7.3) to search and identify putative transcription factor binding sites [65]. PromoterInspector (Genomatix) was used to verify the transcriptional start site (TSS) in the *Notch-2* promoter identified with Gene2Promoter.

The Matrix Family Library Version 7.0 (October 2007) was used and the selected groups were ALL vertebrates.lib where the standard (0.75) core similarity and the optimized matrix similarity was used.

### Plasmids, constructs and cloning of the core and proximal Notch-2 promoter

Full length murine Lef-1 (mLef-1) and human Tcf-4 (hTcf-4) expressed in pCDNA6 were kind gifts from professor B.O Williams while HA-S33Y-β-catenin, expressed in pCGN, was a kind gift from professor Avri Ben-Ze'ev (via professor Anita Sjölander).

For *in vitro* translation experiments HA-S33Y-β-catenin was subcloned into pCDNA3 (Invitrogen, Cambridge, UK) using XbaI and BamHI restriction enzymes.

A larger *Notch-2* luciferase promoter reporter construct (N2PR −2327/−99) (numbering relative to translational start site), covering all four LEF-1/TCF-sites in the *Notch-2* promoter, was constructed using primers:

*N2PR −2327/−99-forward*: 5′-GGTACCTGGGGATTAATAGGCTGTGG-3′

*N2PR −2327/−99-reverse*: 5′- CTCGAGCTCCTGCTTCAAAGGCTCAG-3′,

as well as a smaller (N2PR −110) covering the most proximal LEF-1/TCF-site using primers:

*N2PR −110-forward*: 5′-GGTACCGTTGCACACCCGAGAAAGTT-3′

*N2PR −110-reverse*: 5′- CTCGAGATCTTCTCGGTCGCCTCCT-3′.

Forward primers were designed with an additional KpnI restriction site and reverse primers with a XhoI restriction site. PCR-products were cloned into the pGEM-T vector (Promega) according to manufacturer's conditions and then further cloned into pGL3 luciferase reporter (Promega, Wisconsin, USA) containing a TATA-box (described previously [40]) or pGL3-basic (Promega) using KpnI and XhoI.

*Site-directed mutagenesis of −110 LEF1/TCF site in the Notch-2 promoter*

The plasmid pGL3-basic-N2PR −110 was used as the parental clone for the mutagenesis experiment. A mutation in the −110 LEF1/TCF site was generated by replacing the site with a *SpeI*-restriction site. Briefly, two PCR-amplicons were generated, the first using primers:

*N2PR −110 SpeI-forward*: 5′-AGGAACTAGTAGCAGGAGGAGGGGAGGA-3′ (containing the *SpeI* restriction sequence) and the pGL3-specific primer

GLprimer2: 5′-CTTTATGTTTTTGGCGTCTTCCA-3′, and the second using the pGL3-specific primer RVprimer3: 5′-CTAGCAAAATAGGCTGTCCC-3′ and *N2PR −110 SpeI-reverse*: 5′-CTCGAGGCTAGC ACTAGT GCTCAGGCCCTGGCGCTA-3′ (containing the *SpeI* restriction sequence), with pGL3-TATA-N2PR −110 as a template. Amplicons were separately cloned into the pGEM-T vector and sequentially transferred into pGL3-TATA using *KpnI*, *XhoI* and *SpeI* restriction enzymes thereby mutating the −110 LEF1/TCF core sequence CTTTGA into ACTAGT.

### *In vitro* translation and electrophoretic mobility-shift assay (EMSA)

*In vitro* translated proteins were prepared with the T_N_T® coupled reticulocyte lysate system (Promega). For protein-DNA binding interaction studies, *in vitro* translated Lef-1 and β-catenin were incubated with the following ^32^P-labeled duplex oligonucleotide probe CD1TOP (5′-CTCTGCCGGGCTTTGATCTTTGCTTAACAACA-3′). The binding reaction contained 50 fmol of ^32^P-labeled probe that was incubated for 30 min with the *in vitro* translated proteins in 10 mM Hepes/70 mM KCl/1 mM dithiotreitol/1 mM EDTA/7.5 mM MgCl_2_/4% (vol/vol) glycerol/8 µg/ml salmon sperm DNA. For competition experiments, 300 -or 900-fold excess unlabeled double-stranded oligonucleotides (Probes sequences are found in Table S1), used as competitors, were incubated with the extracts at room temperature 10 min prior to probe addition. Bound complexes were separated on 6% polyacrylamide gels, dried and visualized on X-ray film. T_N_T® coupled reticulocyte lysate without *in vitro* translated proteins was used as a negative control.

### Cell cultivation

HCT116 and HT29 colon cancer cells (ATCC, Manassas, VA, USA) were cultivated in McCoy's 5A media (Gibco/Invitrogen) supplemented with 10% foetal bovine serum at 37°C in 5% CO_2_. Twenty-four hours prior to transfection or chromatin immunoprecipitation, the cells were split with 0.05% Trypsin-EDTA (Gibco/Invitrogen) and counted.

#### ZnCl_2_-induction of wild-type (wt) *Apc* in HT29

HT29 cells harbouring a vector carrying the wt-*Apc* gene (HT29-APC) or the control gene *β-galactosidase* (HT29-β-galactosidase), regulated by a zinc inducible promoter (generous gift from professor B. Vogelstein) were cultivated as described above, with the exception that cells were selectively grown by the addition of 1.2% hygromycine B (Invitrogen). Twenty-four hours before harvest, the cells were split with 0.05% Trypsin-EDTA and counted. HT29-APC and HT29-β-gal. were seeded in MULTIWELL™ 6 well (Falcon, BD Bioscience, San Jose, CA) (1,0×10^6^/well) and 100 µM ZnCl_2_ was added to the medium in the interval of 24, 18, 12, 6, 3 and 0 hours before cell harvest and RNA as well as protein isolation.

#### siRNA treatment of HT29 cells

Cells for siRNA transfection were seeded in MULTIWELL™ 12 well (Falcon) (2.5×10^5^/well), as described above. A pool of four different siRNA oligonucleotides targeting β-catenin mRNA (siGENOME SMARTpool™, Dharmacon, Chicago, IL, USA) with the sequence: GAUCCUAGCUAUCGUUCUU, UAAUGAGGACCUAUACUUA, GCGUUUGGCUGAACCAUCA, GGUACGAGCUGCUAUGUUC, were transfected into HT29 with DharmaFECT4™ transfection reagent (Dharmacon) according to the manufacturer's recommendations. The final siRNA concentration was 100 nM. As control experiment, cells were in parallel mock transfected with siGLO® transfection indicator (Dharmacon) only or a non-specific siRNA pool (siCONTROL™ Non-Targeting siRNA pool, Dharmacon).

### RNA and protein isolation

RNA and protein was isolated with the PARIS™ Kit (Ambion Inc., Austin, TX, USA) according to the supplier's recommendations. The protein concentration was spectrophotometrically measured using Bradford Reagent (Sigma, St. Louis, USA). Briefly, 2 µL sample was mixed with 98 uL Milli-Q H_2_O and 1 mL Bradford Reagent. As standard curve 0/1/2/4/8 1 mg/mL BSA (Sigma) was mixed with Milli-Q H_2_O to a final volume of 100 µL and then 1 mL Bradford reagent was added. The samples were immediately vortexed and 200 µL sample was added to an ELIZA plate and measured by THERMOmax microplate reader (Molecular Devices) with SOFTMAX software (Molecular Devices). All measurements were performed in duplicates.

### cDNA synthesis and semi-quantitative RT-PCR

200 ng total RNA from each sample was reversely transcribed into cDNA with SuperScript™III (Invitrogen) according to the supplier's recommendation. To determine the expression of Apc or silencing of β-catenin, the well-established target gene of an active Wnt pathway, *cyclin D1*, was used as a positive control [9]. As negative controls and controls for equal loading the endogenous genes *Gapdh* was used. For quantitative assessments, the mRNA expression was normalized against *Gapdh*. 1 µL of cDNA solution was added to 19 µL of a standard master mix containing 0.5 units of ThermoWhite DNA Polymerase (Saveen Werner AB, Limhamn, Sweden) and 1 µM of the appropriate primers (Supplemental Information S1 for primer sequences and PCR-conditions). Separation of the PCR products was achived in 1.5% agarose gels and detection with ethidium bromide and UV-light. The software Quantity One (Bio-Rad Laboratories, Hercules, CA, USA) was used for semi-quantitative densiometric analysis.

### Western blot

Cell lysates containing equal amounts of total protein were denatured in NuPAGE® LDS Sample Buffer (Invitrogen) at 70°C for 10 min with NuPAGE® Sample Reducing Agent (Invitrogen). The proteins were separated at 200 V for 35 min in NuPAGE® 4–12% Bis-tris gels (Invitrogen) held in NuPAGE® MES DS Running Buffer (20×) diluted 1∶20 with H_2_O, 0.25% NuPAGE® Antioxidant (Invitrogen) was added to the inner chamber. After the separation, the proteins were blotted to PVDF membranes (Pierce, Rockford, IL, USA) at 30 V for 60 min in NuPAGE® Transfer Buffer (20×) (Invitrogen) diluted 1∶20 in H_2_O with 20% methanol and 0.1% NuPAGE® Antioxidant. The PVDF membranes were blocked with 5% Blotto (Santa Cruz Biotechnology, Inc., Santa Cruz, CA, USA) dissolved in Tris-buffered saline with 0.1% Tween®- 20 (TBS-T) (Sigma) for 60 min at room temperature. The primary antibodies added to the PVDF membranes were diluted in TBS-T as follows: anti-APC 1∶500 (Abcam, Cambridge, UK), anti-β-catenin 1∶1000 (Millipore Corp., Mosheim, France), anti-Gapdh 1∶2000 (Millipore), anti-Notch-2 1∶2000 (Santa Cruz) and anti-His (6× His tag® antibody) 1∶1000 (Abcam). The antibodies were incubated for 60 min at room temperature or over night in 4°C, washed in TBS-T, followed by incubation for 60 min at room temperature with appropriate secondary antibodies, goat-anti-mouse 1∶10000 (Jackson ImmunoResearch Europe Ltd., Suffolk, UK) and goat-anti-rabbit 1∶10000 (Cayman Chemical, Ann Arbor, MI, USA). The PVDF membranes were exposed to ECL Western Blotting Substrate (Pierce) and photographed digitally. Before reincubation of the membranes, the antibodies were stripped with Restore™ Western Blot Stripping Buffer (Thermo SCIENTIFIC, Rockford, IL, USA) for 15 min at room temperature and washed in TBS-T.

### Animals and handling of tumor specimens

C57BL/6 mice with the APC^Min/+^ genotype (The Jackson Laboratory, Bar Harbor, ME, USA) were used in the present study Animals were housed in ventilated cages at 23±1°C with a 12-h light/dark cycle. Standard diet (CRME rodent, Special Diet Services Ltd., Witham, Essex, UK) and water were available *ad libitum*. DNA for genotyping was isolated from tail biopsies with the Extract-N-Amp Tissue PCR Kit™ (Sigma), according to supplier's recommendations. The *Apc* encoding gene were genotyped as previously described [66]. All experimental procedures were approved by the animal Care and Use Committee at the Linköping University (Permit Number: 44-06).

Tumors and non-tumor intestinal mucosa specimens were collected and handled as previously described [67]. mRNA expression of *mNotch-1*, *mNotch-2* and *mHes-1* was subsequently determined with the 7500 Fast Real-Time PCR System (Applied Biosystems, Foster City, CA, USA), using predesigned primer/probe assays purchased from Applied Biosystems (sequences available at request). *C*_t_ values were related to the endogenous control gene GAPDH (Δ*C*_t_), and relative expression (2^−Δ*C*t^) was normalised to the average expression in non-tumor intestinal mucosa of APC^Min/+^mPGES-1^+/+^ mice (2^−ΔΔCt^).

### Luciferase reporter assay

24 hours prior to luciferase reporter assays, HCT116 or HT29 cells were seeded and co-transfected with 200 ng reporter vector (pGL3-TATA with and without N2PR −2327/−99 or −110 insert), 95 ng pSV-β-galactosidase and 455 ng pCGN-HA-S33Y-β-catenin, pcDNA6-mLef-1 or pcDNA6-hTcf-4, using Lipofectamine LTX® according to supplier's recommendations. Controls were co-transfected with empty pCDNA6 vector and 24 h post transfection both luciferase and β-galactosidase activities were measured. As a background control, pGL3-TATA without insert was used and treated as described above. The luciferase activity was determined by the ratio of luciferase to β-galactosidase activity and the relative activity by the ratio of promoter activity vs. background control.

### Chromatin immunoprecipitation

To investigate binding of LEF-1/TCF-family members Lef-1 and Tcf-4 to *Notch-2* LEF-1/TCF-site −110, chromatin immunoprecipitation (ChIP) assays were carried out using a ChIP Assay Kit (Millipore). Briefly, 2.5×10^5^ HCT116 cells/well were seeded in MULTIWELL™ 6 well (Falcon) and co-transfected with 200 ng pGL3-TATA-N2PR −2327/−99, 200 ng pGL3-TATA-N2PR −110 and either 2.1 µg 6×histidine tagged pcDNA6-hTcf-4 or pcDNA6-mLef-1, respectively, using Lipofectamine LTX (Invitrogen). 48 h post transfection, cells were fixed with 1% formaldehyde for 10 minutes and washed twice in ice-cold phosphate-buffered saline. Cells were lysed in sodium dodecyl sulfate lysis buffer (1% sodium dodecyl sulfate, 10 mM EDTA, 50 mM Tris-HCl [pH 8.0]) containing protease inhibitors, and DNA in the cross-linked chromatin preparations was sonicated to an average fragment size of 0.2–1.0 kb using a Bioruptor™ (Diagenode, NJ, USA) and 14 pulses for 30 seconds sonication followed by 30 seconds of rest. The insoluble material was removed by centrifugation, and soluble chromatin samples were precleared with a 50% slurry of protein A-Sepharose-salmon sperm DNA. Each sample was incubated overnight at 4°C with 5 µg of rabbit polyclonal antibodies 6× His tag® antibody - ChIP Grade (Abcam). As a negative isotype control, Normal rabbit IgG (Millipore) was used. Immune complexes were collected with protein A-Sepharose and eluted. Input templates were purified from 5% of the original lysates in parallel with the eluted immunoprecipitated samples. Cross-linking was reversed by incubation at 65°C for 4 h in 200 mM NaCl. After phenol-chloroform extraction and ethanol precipitation, the recovered DNA (2 µl from immunoprecipitated chromatin DNA samples or 2 µl from the input DNA control) was subjected to PCR amplification with the following primers:

*N2PR −110 ChIP forward*: 5′- GGGGAGTCGAGGCATTTG -3′

*N2PR −110 ChIP reverse*: 5′- AGGAGCCCCACTCTCTCCT -3′.

As a negative control 170 bp of the NCAPD2 (non-SMC condensin I complex, subunit D2) were amplified using primers:

*NCAPD2 ChIP forward*: 5′-ATGGTTGCCACTGGGGATCT-3′

*NCAPD2 ChIP reverse*: 5′-TGCCAAAGCCTAGGGGAAGA-3′.

### Statistic, graphs and image handling

All statistics and graphs are computed in GraphPad Prism 5 (GraphPad Software Inc., San Diego, CA, USA) or SPSS v15.0 (SPSS UK Ltd, Woking, UK). Group comparisons for the *in vitro* luciferase assays were performed using Independent sample t-tests and error bars describe the standard error of the mean (SEM). Differences in mRNA levels between mouse adenomas and normal murine intestinal tissue were analyzed with the Mann-Whitney *U*-test (non-normal distributed data) and error bars describe the interquartile range. Figure composition and adjustments in whole image contrast levels were performed in Adobe Photoshop CS4.

## Supporting Information

Figure S1 **Competitive electro mobility-shift assay reveals binding of** ***in vitro*** **translated β-catenin/Lef-1 to** ***Notch*** **pathway gene promoters.** Duplex CD1TOP probes were “end labeled” with [^32^P]dATP, incubated with *in vitro* translated Lef-1/β-catenin and exposed to competition with abundance of cold duplex oligonucleotides (×300 and ×900, respectively). The protein-DNA complexes were separated by electrophoresis and visualized by autoradiography. As a competition control cold CD1TOP and cold mutated CD1FOP competed with radiolabeled CD1TOP and to confirm that radioactive labeled CD1TOP binds Lef-1 specifically, plasmid-free reticulocyte lysate were subjected to *in vitro* translation and incubated with radiolabeled probe. Competition of cold and –cold mutated *Jagged-1* −1933, −1635, −1083, −238, *Maml-1* −746, −698, −1864 and *Hes-1* −528, −568, −476, Positive competitor control, plasmid free reticulocyte lysate, *Notch-2* −2261, −869, −689, −110, *Rfng* −2481, *Numb* −342, *Lfng* −2128, −1513, −1291, −1023 and *Numbl* −2587, −2480 and −1919. Numbering of putative LEF-1/TCF-sites is relative to each gene's translational start site.
(TIF)

Figure S2 **Notch pathway gene expression following** ***RNAi*** **silencing of β-catenin in HT29 cells.** Gene expression was normalized against the negative control *Gapdh*. The expression is presented 0–96 h post transfection. (A) The expression of *Cyclin D1*, *Gapdh*, *Hes-1*, *Hes-7*, *Notch-2*, *Maml-1*, *Lfng*, *Rfng*, *Numb* land *Numb*. (B) The protein expression of β-catenin (∼88 kDa) and loading control, Gapdh, 0–96 h post transfection of anti-β-catenin-siRNA.
(TIF)

Figure S3 **β-catenin and** ***cyclin D1*** **expression remain unaffected in DAPT treated HT29 cells.** HT29 cells were treated with 12.5 µM of the γ-secretase inhibitor DAPT for 24 h thereby inhibiting Notch signaling. (A) Western blots with Hes-1 and β-catenin antibodies on HT29 whole-cell lysate following 24 h DAPT treatment. *Gapdh* was used as a loading control. (B) The expression of *cyclin D1* was semi-quantitatively determined in DAPT treated HT29-cells. Semi-quantitative RT-PCR was carried out on cDNA reversely transcribed from 200 ng total RNA. Bars describe the relative expression of *cyclin D1* in HT29 normalized against *Gapdh* expression.
(TIF)

Figure S4 HT29 cells were co-transfected with pGL3-TATA carrying N2PR -110 (or empty pGL3-TATA) and HA-S33Y-β-catenin in pCGN as well as pSV-β-galactosidase control vector (n = 6).
(TIF)

Table S1 (XLS)

Supplemental Information S1 (DOC)
