# Supplementary figures and images for: The Notch-2 Gene Is Regulated by Wnt Signaling in Cultured Colorectal Cancer Cells
Source: PLoS One. 2011 Mar 18;6(3):e17957. doi: 10.1371/journal.pone.0017957 (PMC3060910; doi:10.1371/journal.pone.0017957)

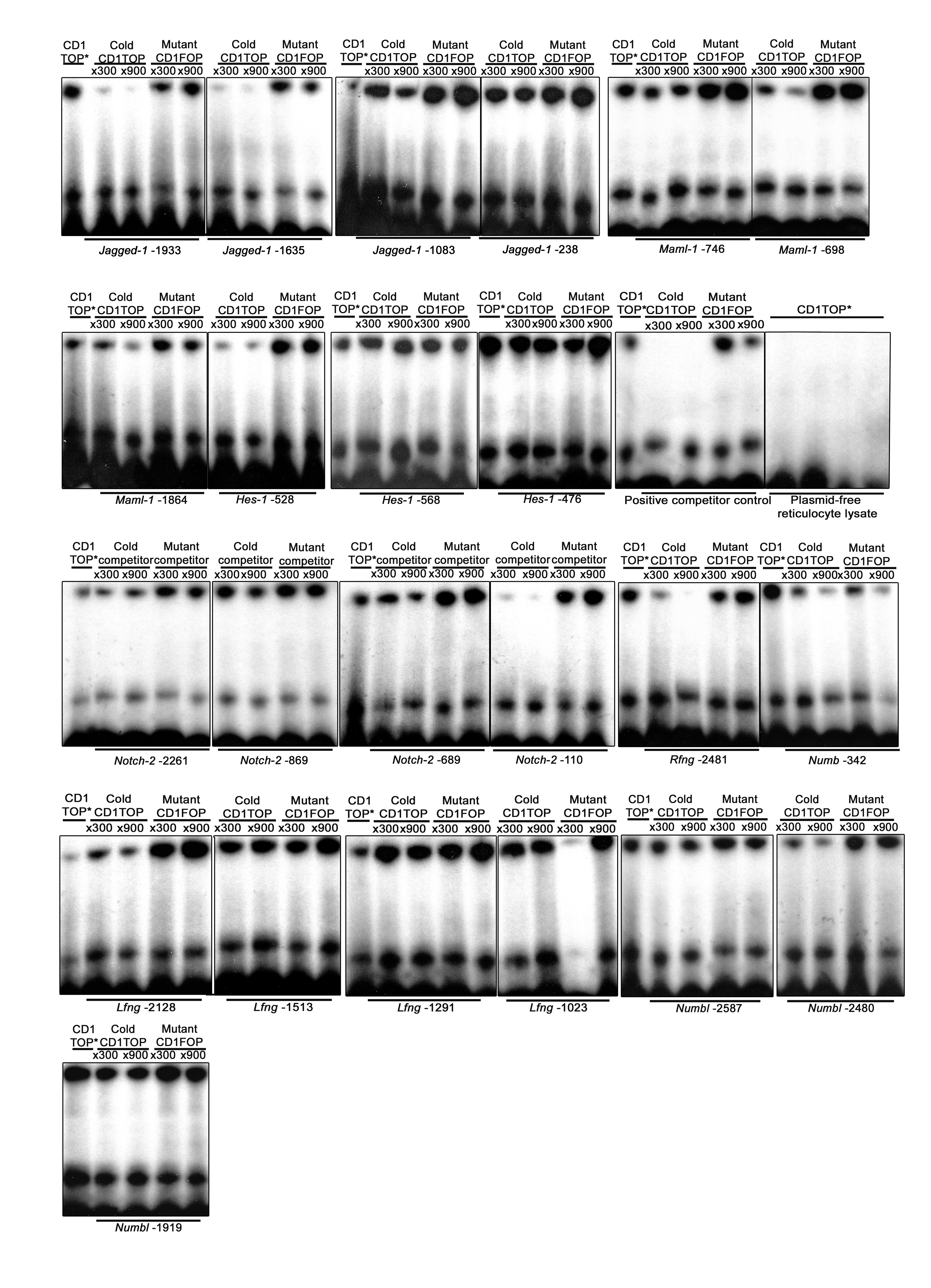

Supplement: Figure S1 — Competitive electro mobility-shift assay reveals binding of in vitro translated β-catenin/Lef-1 to Notch pathway gene promoters. Duplex CD1TOP probes were “end labeled” with [32P]dATP, incubated with in vitro translated Lef-1/β-catenin and exposed to competition with abundance of cold duplex oligonucleotides (×300 and ×900, respectively). The protein-DNA complexes were separated by electrophoresis and visualized by autoradiography. As a competition control cold CD1TOP and cold mutated CD1FOP competed with radiolabeled CD1TOP and to confirm that radioactive labeled CD1TOP binds Lef-1 specifically, plasmid-free reticulocyte lysate were subjected to in vitro translation and incubated with radiolabeled probe. Competition of cold and –cold mutated Jagged-1 −1933, −1635, −1083, −238, Maml-1 −746, −698, −1864 and Hes-1 −528, −568, −476, Positive competitor control, plasmid free reticulocyte lysate, Notch-2 −2261, −869, −689, −110, Rfng −2481, Numb −342, Lfng −2128, −1513, −1291, −1023 and Numbl −2587, −2480 and −1919. Numbering of putative LEF-1/TCF-sites is relative to each gene's translational start site. (TIF) [file pone.0017957.s001.tif]

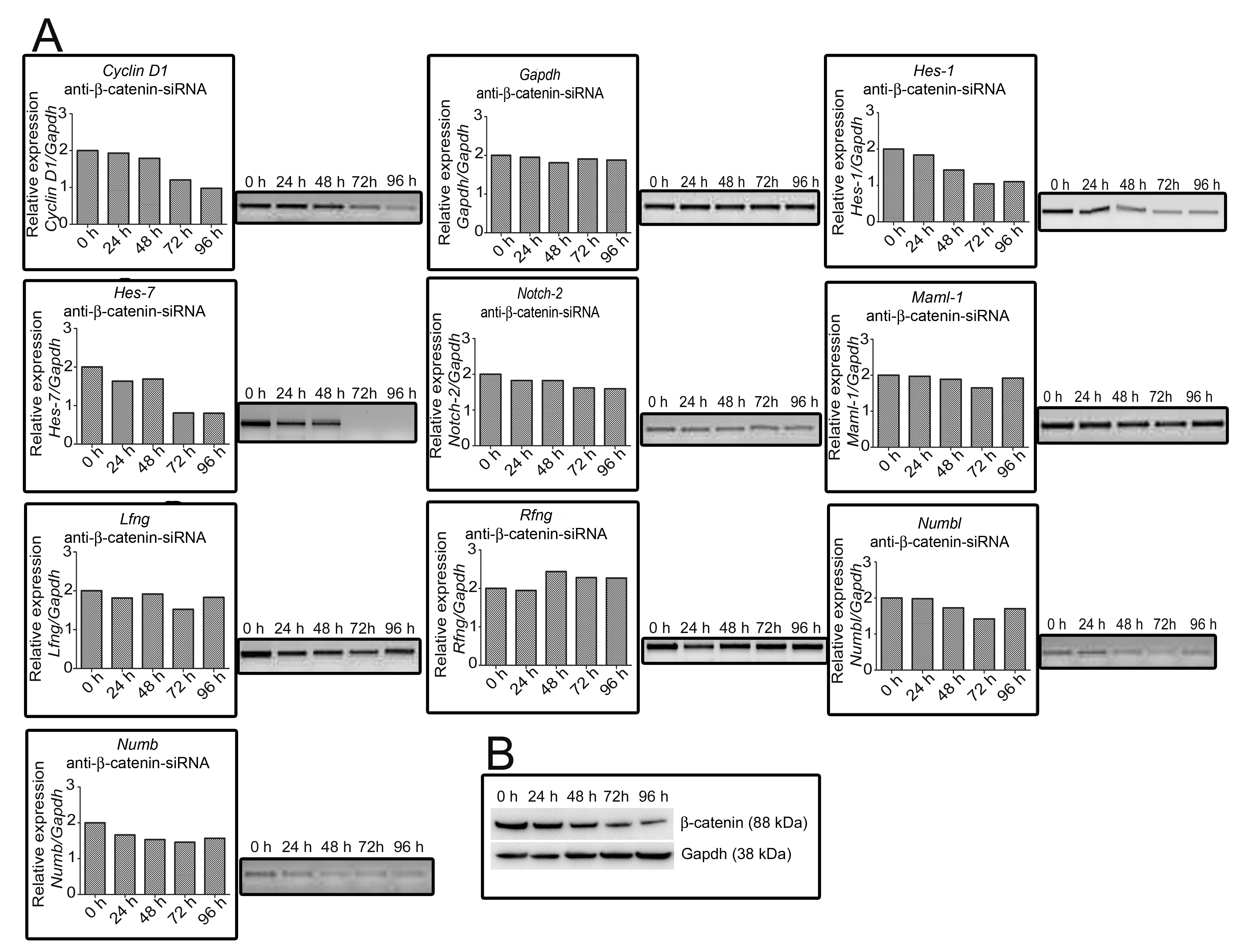

Supplement: Figure S2 — Notch pathway gene expression following RNAi silencing of β-catenin in HT29 cells. Gene expression was normalized against the negative control Gapdh. The expression is presented 0–96 h post transfection. (A) The expression of Cyclin D1, Gapdh, Hes-1, Hes-7, Notch-2, Maml-1, Lfng, Rfng, Numb land Numb. (B) The protein expression of β-catenin (∼88 kDa) and loading control, Gapdh, 0–96 h post transfection of anti-β-catenin-siRNA. (TIF) [file pone.0017957.s002.tif]

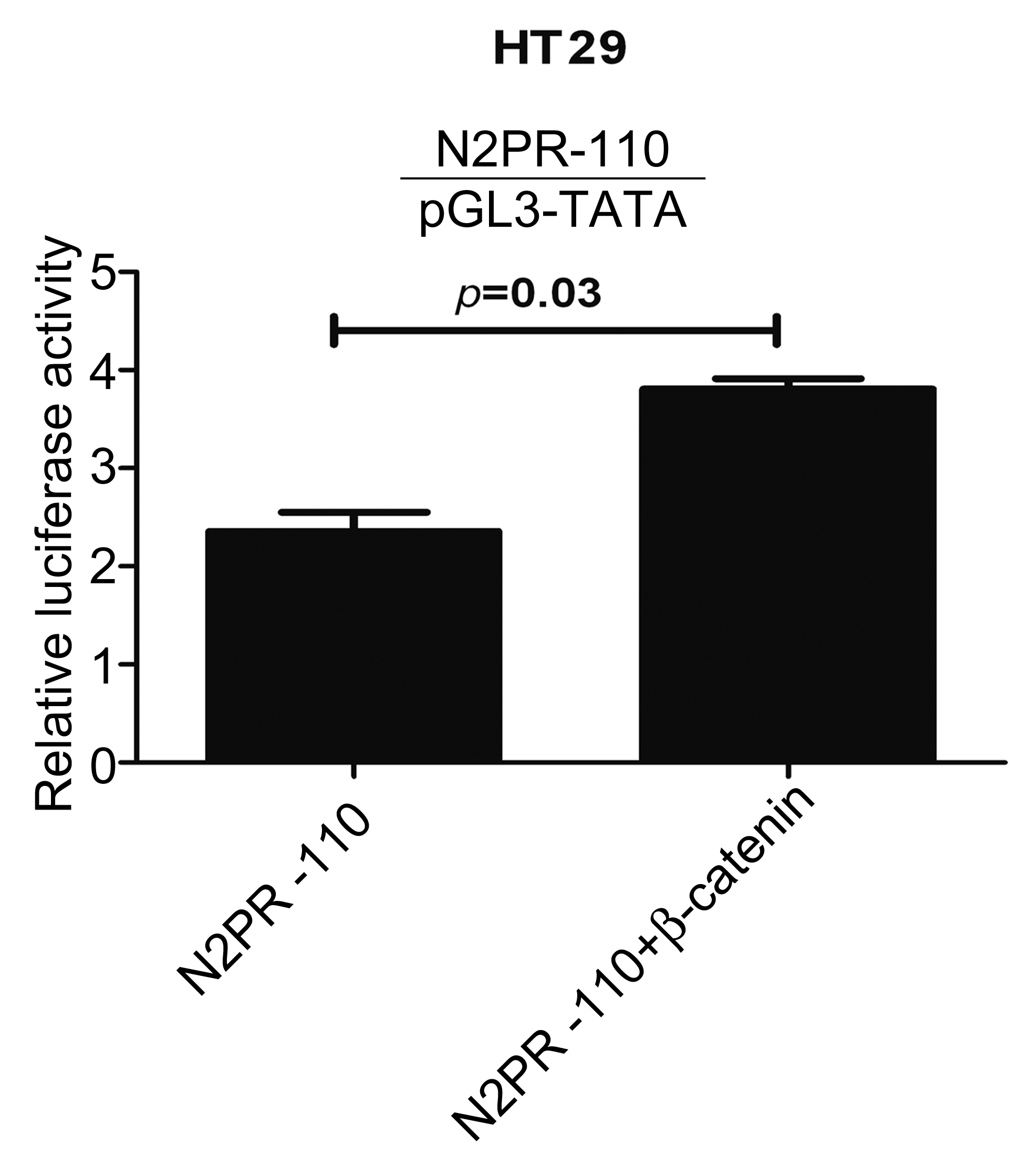

Supplement: Figure S3 — β-catenin and cyclin D1 expression remain unaffected in DAPT treated HT29 cells. HT29 cells were treated with 12.5 µM of the γ-secretase inhibitor DAPT for 24 h thereby inhibiting Notch signaling. (A) Western blots with Hes-1 and β-catenin antibodies on HT29 whole-cell lysate following 24 h DAPT treatment. Gapdh was used as a loading control. (B) The expression of cyclin D1 was semi-quantitatively determined in DAPT treated HT29-cells. Semi-quantitative RT-PCR was carried out on cDNA reversely transcribed from 200 ng total RNA. Bars describe the relative expression of cyclin D1 in HT29 normalized against Gapdh expression. (TIF) [file pone.0017957.s003.tif]

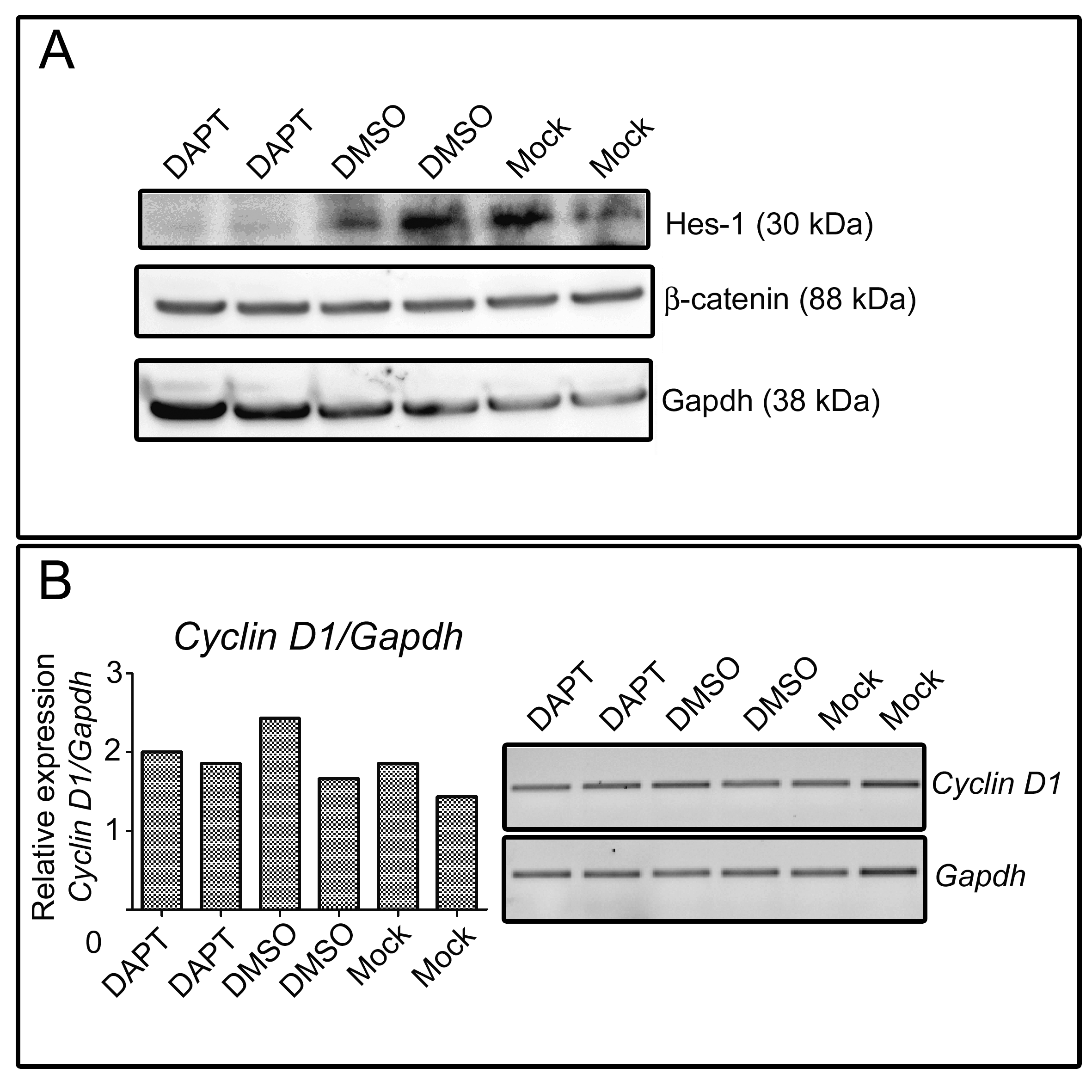

Supplement: Figure S4 — HT29 cells were co-transfected with pGL3-TATA carrying N2PR -110 (or empty pGL3-TATA) and HA-S33Y-β-catenin in pCGN as well as pSV-β-galactosidase control vector (n = 6). (TIF) [file pone.0017957.s004.tif]
