# Supplementary material for: The Notch-2 Gene Is Regulated by Wnt Signaling in Cultured Colorectal Cancer Cells
Source: PLoS One. 2011 Mar 18;6(3):e17957. doi: 10.1371/journal.pone.0017957 (PMC3060910; doi:10.1371/journal.pone.0017957)
Supplement: Supplemental Information S1 — (DOC) [file pone.0017957.s006.doc]

**Supplementals: *Notch-2* and the Notch signaling pathway are regulated by Wnt signaling in colorectal cancer cells**

***Table S2:*** *In silico analysis of Notch and Wnt pathway gene promoters.*

| ***Notch pathway genes*** | ***LEF-1/TCF*** | ***Wnt pathway genes*** | ***RBP-Jκ*** |
| --- | --- | --- | --- |
| ***Jagged-1*** | 4 | *Frizzled 3* | 9 |
| ***Deltalike-1 (Dll1)*** | 3 | *Frizzled 4* | 2 |
| ***Hes-7*** | 2 | *Frizzled 6* | 2 |
| ***Deltalike-3 (Dll3)*** | 3 | *SFRP 1* | 1 |
| ***Deltalike-4 (Dll4)*** | 1 | *SFRP 2* | 1 |
| ***Numb*** | 1 | *SFRP 3* | 2 |
| ***Numbl*** | 4 | *SFRP 4* | 2 |
| ***Hey-2*** | 6 | ***Apc*** | 12 |
| ***Heyl*** | 2 | *DVL-2* | 3 |
| ***Fringe lunatic (Lfng)*** | 4 | *Axin-1* | 6 |
| ***Fringe radical (Rfng)*** | 1 | *Axin-2* | 7 |
| ***Fringe manic (Mfng=*** | 5 | *GSK-3β* | 2 |
| ***Maml-1*** | 5 | *CK1* | 3 |
| ***Notch-2*** | 4 | *LRP5* | 2 |
| ***Notch-3*** | 1 | *LRP6* | 1 |
| ***Notch-4*** | 1 | *Bcl9-2* | 5 |
| ***Hes-1*** | 3 | *HDAC 2* | 1 |
| ***Hes-3*** | 2 | *Beta-TrCP* | 1 |
| ***Hes-5*** | 1 | *Wnt-1* | 5 |
| *RBP-Jκ* | 5 | *Wnt-2* | 1 |
| *SHARP* | 2 | *Wnt-3a* | 2 |
| *Gamma secretase APH-1A* | 3 | *Wnt 4* | 3 |
| *Gamma secretase APH-1B* | 2 | *Wnt-5a* | 7 |
| *Gamma secretase PEN2* | 2 | *Wnt-5b* | 11 |
|  |  | *Wnt-6* | 2 |
|  |  | *Wnt-9a* | 2 |
|  |  | *Asef* | 13 |
|  |  | *Dkk-1* | 3 |

**Legend Table S2:** In silico analysis of Notch and Wnt pathway gene promoters identifies several LEF-1/TCF sites in the Notch pathway and Rbp-jκ in the Wnt pathway indicating a direct crosstalk via target gene activation. Genes highlighted in bold was further investigated with semi quantitative PCR or Western blot.

**Table S3: Notch pathway gene primers for semi-quantitative PCR.**

| ***Gene*** | ***Forward primer*** | ***Reverse primer*** | ***T_m_*** | ***Cycles*** |
| --- | --- | --- | --- | --- |
| ***Notch-2*** | 5’-TCAGGGGTTAATTGTGAAAT-3’ | 5’-ATATACAGCGGAAACCATTC-3’ | 55°C | 30 |
| ***Notch-3*** | 5’- TGTGGACGAGTGCTCTATCG -3’ | 5’- AATGTCCACCTCGCAATAGG-3’ | 55°C | 35 |
| ***Notch-4*** | 5’- CAGAGGGCTGCTGTGTGGGA -3’ | 5’- GAGAGGGAGAGCTGGGGAGC-3’ | 60°C | 35 |
| ***Deltalike-1*** | 5’- GGGTGGAGAAGCATCTGAAA -3’ | 5’- TGGGGCATATATCCTTGGAA -3’ | 58°C | 40 |
| ***Deltalike-3*** | 5’- GAGCTGCGCTTCTCGTACC -3’ | 5’- TCAAAGGACCTGGGTGTCTC -3’ | 60°C | 30 |
| ***Deltalike-4*** | 5’- TATTGGGCACCAACTCCTTC -3’ | 5’- AACCAGTTCTGACCCACAGC -3’ | 60°C | 40 |
| ***Hes-1*** | 5’-GCATCTGAGCACAGAAAGTC -3’ | 5’- GAGGTGCTTCACTGTCATTT -3’ | 55°C | 38 |
| ***Hes-3*** | 5’- ACAGGGACTATTTTCAGCAC -3’ | 5’- TGTAGGTGGAGCTAGAGTGG -3’ | 60°C | 35 |
| ***Hes-5*** | 5’- GACTGCGGAAGCCGGTGGTG -3’ | 5’- CTGGTGCAGGCTCTTGGGGC -3’ | 55°C | 35 |
| ***Hes-7*** | 5’-CTGGAAGAGCTGAGGCTGCT -3’ | 5’-GGTTTGGGGCGCAGATAG -3’ | 55°C | 38 |
| ***Hey-2*** | 5’- CAACATCTCAGATTATGGCAAGA -3’ | 5’- TTGCCTGAAGCATCTTCAAAT -3’ | 55°C | 40 |
| ***Heyl*** | 5’- AGATGCAAGCCAGGAAGAAA -3’ | 5’- GGCATGGAGCATTTTCAAGT -3’ | 55°C | 35 |
| ***Fringe lunatic (Lfng)*** | 5’-ACGTCTACGTCGGCAAGC -3’ | 5’-AGGTTCTCCAGGTGGGAGTG -3’ | 55°C | 38 |
| ***Fringe radical (Rfng)*** | 5’- ACCCAGCCAGGACGTCTAC -3’ | 5’- CCTCCACGATGTAGCCAACT -3’ | 55°C | 38 |
| ***Fringe manic (Mfng)*** | 5’- CCATGTGGACGATGACAACT -3’ | 5’- TATAGCCCATGGTGCAGTCA -3’ | 55°C | 35 |
| ***Mastermind-like-1 (Maml-1)*** | 5’- ACAGGGTCCTCTGCTGCCGT -3’ | 5’- AGCCACACCCCGGTCCTGTT -3’ | 55°C | 30 |
| ***Numb*** | 5’- AAAATGCAGTGCCAGAAGTA -3’ | 5’- GAAGGTAGGAGATTGTGGTG -3’ | 60°C | 30 |
| ***Numbl*** | 5’-GCGACAGTGACAGCATCAAC | 5’- CTCAGCCTCTGAAGGTGTCC -3’ | 60°C | 30 |
| ***Hath-1*** | 5’-CCGCCCAGTATTTGCTACAT-3’ | 5’-CATTCACCTGTTTGCTGGAA-5’ | 55°C | 35 |
| ***GAPDH*** | 5’-GAGTCAACGGATTTGGTCGT -3’ | 5’- GACAAGCTTCCCGTTCTCAG -3’ | 60°C | 22 |
| ***Cyclin D1*** | 5’- AACTACCTGGACCGCTTCCT -3’ | 5’- CCACTTGAGCTTGTTCACCA -3’ | 55°C | 30 |

***Table S4:*** *Comparison of relative luciferase activity for N2PR -2327/-99 and N2PR -110 co-transfected with S33Y-β-catenin, hTcf-4 or mLef-1in HCT116 cells.*

| *Constructs* | *Relative luciferase activity (mean vs. mean (±SEM), n=6)* | *Fold increase* | *P-value* |
| --- | --- | --- | --- |
| N2PR -2327/-99+Tcf-4 (b) vs. N2PR -2327/-99 (a) | 35.0 (±3.5) vs. 17.5 (±5.0) | 2.0 | 0.02 |
| N2PR -2327/-99 +Lef-1 (c) vs. N2PR -2327/-99 (a) | 49.0 (±4.6) vs. 17.5 (±5.0) | 2.8 | 0.001 |
| N2PR -2327/-99+β-catenin (d) vs. N2PR -2327/-99 (a) | 90.6 (±14.3) vs. 17.51 (±5.0) | 5.2 | 0.0007 |
| N2PR -110+Tcf-4 (f) vs. N2PR -110 (e) | 2.0 (±0.2) vs. 2.2 (±0.2) | 0.9 | n.s |
| N2PR -110+Lef-1 (g) vs. N2PR -110 (e) | 2.7 (±0.2) vs. 2.2 (±0.2) | 1.2 | n.s |
| N2PR -110+β-catenin (h) vs. N2PR -110 (e) | 4.0 (±0.2) vs. 2.2 (±0.2) | 1.8 | <0.0001 |
| N2PR -2327/-99+Tcf-4 (b) vs. N2PR -2327/-99+Lef-1 (c) | 35.0 (±3.5) vs. 49.0 (±4.6) | 1.4 | 0.04 |
| N2PR -2327/-99+β-catenin (d) vs. N2PR -2327/-99+Tcf-4 | 90.6 (±14.3) vs. 35.0 (±3.5) | 2.6 | 0.004 |
| N2PR -2327/-99+β-catenin vs. (d)N2PR -2327/-99+Lef-1 (c) | 90.6 (±14.3) vs. 49.0 (±4.6) | 1.8 | 0.02 |
| N2PR -110+Lef-1 (g) vs. N2PR -110+Tcf-4 (f) | 2.7 (±0.2) vs. 2.0 (±0.2) | 1.4 | 0.005 |
| N2PR -110+β-catenin (h) vs. N2PR -110+Tcf-4 (f) | 4.0 (±0.2) vs. 2.0 (±0.2) | 2.0 | <0.0001 |
| N2PR -110+β-catenin (h) vs. N2PR -110+Lef-1 (g) | 4.0 (±0.2) vs. 2.7 (±0.2) | 1.5 | 0.0001 |
